# Rapid response of moss-associated nitrogen fixation to nutrient additions in tropical montane cloud forests with different successional stages

**DOI:** 10.1007/s10533-024-01195-3

**Published:** 2025-01-04

**Authors:** Lina Avila Clasen, Danillo Oliveira Alvarenga, Yinliu Wang, Rune Fromm Andersen, Kathrin Rousk

**Affiliations:** 1 Department of Biology, University of Copenhagen, Universitetsparken 15, 2100 Copenhagen, Denmark; 2 Center for Volatile Interactions, University of Copenhagen, Universitetsparken 15, 2100 Copenhagen, Denmark

**Keywords:** Nitrogen fixation, Mosses, Forest succession, Tropics, Phosphorus, Molybdenum

## Abstract

Nitrogen (N) fixation in association with mosses could be a key source of new N in tropical montane cloud forests since these forests maintain high humidity levels and stable temperatures, both of which are important to N fixation. Here, nutrient availability could be a prominent control of N fixation processes. However, the mechanisms and extent of these controls, particularly in forests at different successional stages, remains unknown to date. To address this knowledge gap, we investigated the impact of N, phosphorus (P) and molybdenum (Mo) additions on moss-associated N fixation in tropical montane cloud forests of two successional stages, an old-growth forest and an early-successional natural regrowth forest. We hypothesized that if N is available, N fixation rates would be rapidly reduced, while P and Mo would promote nitrogenase activity. Our results show that Mo additions did not affect N fixation rates, whereas N and P additions, in different doses and combinations, immediately reduced N fixation in both forests. Nonetheless, rates recovered within 1 year of nutrient additions. Nitrogen fixation rates associated with ground-covering mosses were similar in both forests. Interestingly, one year after the nutrient additions, N fixation rates across all the treatments were higher in the natural regrowth forests than the mature forests, suggesting more nutrient limitation in these regrowing forests, likely as a result of higher demand for growth. Our study highlights how moss-associated N fixation responds to changes in nutrient availability across distinct successional stages, deepening our understanding of processes that contributes to tropical montane cloud forests.

**Supplementary Information:**

The online version contains supplementary material available at 10.1007/s10533-024-01195-3.

## Introduction

Nitrogen (N) is one of the main nutrients influencing biodiversity, composition and productivity of ecosystems (Vitousek et al. 2010; Du et al. 2020). Although the atmosphere is primarily composed of N gas (N_2_), that N is not directly accessible by plants. In terrestrial ecosystems, N-fixing bacteria present in the soil or in association with plants are largely responsible for reducing N_2_ to ammonia (NH_3_) via the nitrogenase enzyme complex (Bellenger et al. 2020), which can then be absorbed by plants and other organisms. In natural ecosystems, it is estimated that 80–90% of the N available to plants can be derived from biological N fixation (Rascio and La Rocca 2018). The contribution by free-living N fixation is estimated to reach higher means in tropical regions (e.g. tropical evergreen forests 7.8 (0.1–60) kg ha^−1^ yr^−1^), opposed to ~ 1.5 (0.3–3.8) kg ha^−1^ yr^−1^ in tundra and boreal forests, where the majority of studies investigating moss-associated N fixation have been conducted (Reed et al. 2011). Globally, N fixation in association with bryophytes, lichens, soil crusts and others can contribute for nearly half (49 [27–99] Tg N yr^−1^) of the biological N fixation in terrestrial ecosystems, significantly impacting terrestrial CO_2_ sequestration (Elbert et al. 2012).

In bryophytes, the N-fixing bacteria most often found are symbiotic cyanobacteria, which are among the largest contributors to new N in natural ecosystems like forests, peatlands and tundras (Alvarenga and Rousk 2022). In boreal forests and subarctic regions, a large number of studies have focused on mosses and their associated N-fixing bacteria, showing that they can contribute between 1.5 and 2.2 kg N ha^−1^ yr^−1^—similar rates that enter these systems via N deposition (DeLuca et al. 2002; Gundale et al. 2011; Ackermann et al. 2012; Rousk et al. 2013; Holland-Moritz et al. 2018). The potential of mosses to provide N via N fixation was previously observed in both lowland and montane tropical forests. These forests generally show differences regarding nutrient limitation—with montane forests often pointed as more N limited (Gay et al. 2022). A study in Puerto Rico investigating both low and upper montane forests observed higher nitrogenase activity associated with mosses compared to soil and litter, with the contribution by mosses more significant in the upper forests (Cusack et al. 2009). A higher contribution to forest N fixation by epiphytic bryophytes vs. soil and litter was also observed in a montane cloud forest in Costa Rica (Alvarenga et al. 2024). Here, N fixation rates per mass basis in association with epiphytic bryophytes were 57 times higher than the ones found in litter, and 270 times of those measured in soil, with light driving N fixation rates in the epiphytes. Most recently, high contributions via moss-associated N-fixers have also been estimated in tropical montane cloud forests in Peru with the contribution reaching 2 kg N ha^−1^ yr^−1^ (Permin et al. 2022). Another study conducted in a high-elevation cloud forest in Costa Rica has estimated the contribution of bryophytes to be of 6 (± 1.9) kg N ha^−1^ yr^−1^, 28% of which was attributed to moss-associated N fixation (Markham and Fernández Otárola 2021). The potentially high contribution of new N coming from those associations to heterogeneous tropical ecosystems calls for more research to understand the controls of N fixation in habitats facing climate change and other ecosystem disturbances.

Environmental factors, like temperature and moisture, are often pointed out as main regulators of N fixation in association with bryophytes, soil, litter and other cryptic sources, with nutrient availability being pointed as the second most important factor affecting the process (Cleveland et al. 2022). As tropical montane cloud forests are characterized by high relative humidity and the ability to uphold a relatively constant temperature beneath the forest canopy (Bonan 2008), moisture and temperature could be less limiting in those forests. This suggests that nutrient availability could play a bigger role in regulating N fixation in these tropical ecosystems than previously thought.

The theoretical cost associated with the reduction of one molecule of N_2_ to ammonia is 16 ATPs. Under natural conditions, the actual cost may surpass this theoretical value, primarily due to the sub-optimal efficiency of the process (Rascio and La Rocca 2018). Nevertheless, in the presence of other available forms of N, this process can be rapidly reduced or become temporally inactive (Rousk et al. 2013; Bellenger et al. 2020; Alvarenga and Rousk 2021; Clasen et al. 2023). However, nitrogenase activity can be re-established when the system become once more N-limited, with recovery varying with time and N source (Wang et al. 2022). On the other hand, phosphorus (P), can limit N fixation, especially in tropical forests when compared to other biomes (Dynarski & Houlton 2018). Given the high ATP expense of the process and the crucial role of P as an energy source for nitrogenase reactions and cell metabolism, P can also limit plant growth (Reed et al. 2011). A limitation by P seems intuitive, with higher availability of P possibly leading to N-limitation and thereby promotion of nitrogenase activity. However, contrasting results have been reported from different tropical forest compartments. For instance, no response to P additions were found in either lowland tropical soils (Wurzburger et al. 2012) or southern Amazonian soil and litter (Wong et al. 2021). In contrast, promotion of N fixation rates by P additions in soil and litter was seen in other lowland tropical forests in French Guiana (Van Langenhove et al. 2021). Another lab-based study with tropical mosses also suggests possible promotion of N fixation by P additions, although results were variable and possibly dependent on species and time after P additions (Clasen et al. 2023). This can be due to the fact that bryophytes, including mosses, can differ in resource acquisition and storing of nutrients (Liu et al. 2020; Fan et al. 2022). Besides, promotion by P can take weeks or months to be observed as it is likely connected to growth and photosynthesis of both mosses and associated cyanobacterial community that in turn can increase N fixation rates (Rousk et al. 2017). On the other hand, P combined with N additions, resulted in enhanced N fixation rates in Hawaiian soils by alleviating P limitations, which is crucial for the energy-intensive process (Matson et al. 2015).

Besides P, molybdenum (Mo) as a key element and co-factor of the most common form of the nitrogenase enzyme can promote N fixation (Silvester 1989). However, although Mo additions can lead to higher N fixation rates in some tropical lowland soils (Barron et al. 2009; Van Langenhove et al. 2021), and in association with subarctic mosses (Rousk et al. 2017), they do not always lead to promotion either, as shown in some Amazonian soils (Wong et al. 2021). Further research is needed on the factors controlling N fixation and the role of nutrients in tropical bryophytes. These, are widely distributed across tropical ecosystems and are exposed to diverse environmental conditions and nutrient pools, particularly in forests with varying land use histories. Besides, terrestrial bryophytes grow, to a level, detached from soil nutrient pools, which likely leads to stronger nutrient limitation compared to vascular plants as bryophytes are dependent on nutrient input also from deposition and throughfall, with environmental conditions also affecting their stoichiometry (Liu et al. 2020; Fernández-Martínez et al. 2021). This in turn leads to higher sensitivity towards fluctuation in nutrient input. That also occurs in secondary forests, which undergo most changes in nutrient availability and biogeochemical processes in the first years of forest regrowth, and changes in N availability are some of the major shifts driving communities with N limitation typically declining throughout succession (Powers and Marín-Spiotta 2017), which can also affect N fixation rates, with the process possibly contributing to the regeneration of younger forests.

Nitrogen fixation associated with mosses, as well as in soil and litter, increased with forest age in forests (with different vegetation) in a work conducted in southern China, although neither N or P substrate content could explain the variation in N fixation (Zheng et al. 2020), which sounds counterintuitive since higher availability of N should decrease N fixation processes. Another study investigating N fixation also associated to bryophytes in Hawaiian rainforests, found similar rates across different soil-age gradient forests even though soil nutrient content varied across forests (Matzek and Vitousek 2003). In addition, research from temperate forests in New Zealand indicate that bryophytes could be decoupled from soil N in those forests, and although the authors found generally high rates associated with bryophytes in younger soil sites, poor in N but with high P availability, their final contribution tends to be higher in the intermediate and older forests when total forest biomass is taken into account (Menge and Hedin 2009). Nevertheless, further investigation is still needed to unravel these different responses, especially in tropical montane cloud forests, to understand the controls of N fixation in association with mosses in these unique ecosystems.

In this study, we aimed to answer some of the open questions regarding the effects of nutrients on N fixation in association with tropical montane mosses. For that purpose, we selected two forest types in central Costa Rica, an old growth montane cloud forest and an early-succession natural regrowth forest of approximately 15 years old. Here, we investigated the response of N fixation rates to N, P, NP and Mo additions and different collection times (1 day and 1 year after the first addition, 1 day after the second addition) to assess both immediate effects and a possible delayed response to the nutrients added. We hypothesized that N fixation rates are higher in association with mosses from the younger forest sites (natural regrowth) (H1), although higher abundance and biomass of mosses would be observed in the older forests compared to the younger one. We also hypothesized that the addition of inorganic N would rapidly reduce N fixation rates (H2a), although this reduction would be alleviated when P was applied in combination with N (H2b). Moreover, P additions would generally promote N fixation rates, with higher promotion linked to the higher concentrations of applied P and time after application (H3). Similarly, Mo additions would rapidly and positively affect moss-associated N fixation rates (H4).

## Methods

### Study sites

The two forests are located at the Cloudbridge Nature Reserve, bordering the Chirripó National Park in Costa Rica. The reserve was initially established for regeneration and conservation purpose of the surrounding tropical oak montane cloud forests of the Cordillera de Talamanca in which both the reserve and the national park are included. The climate comprises a wet season (usually from May to November) and a dry season (usually from December to April), with the average relative air humidity mostly higher than 85% (Kapelle and van Uffelen 2006). Inside the reserve, we selected two forests with different conservation status: (1) an old growth (OG) forest at 1950 m altitude (9° 27′ 58.8′′ N 83° 34′ 18.4′′ W), highly diverse and densely vegetated; and (2) a natural regrowth (NR) forest at 1650 m (9° 28′ 12.8′′ N 83° 34′ 37.0′′ W), formerly used for agriculture purposes. The natural regrowth forest was left to regenerate since 2008, therefore its vegetation is much younger and less dense, with higher light and fewer mature trees (Fig. S1).

Microclimate sensors (TMS-4-TOMST, Prague, Czechia) were installed in both forests recording soil (−6 cm) and air temperatures (+2 and +15 cm), as well as soil moisture, measuring every 15 min for ~ 13 months (Nov 2021–Jan 2022, Fig. S2). Volumetric soil moisture were obtained through conversion of raw dialectic permittivity counts from sensors in the field using a generic equation for high organic matter soils (Wild et al. 2019). Light was measured using a photosynthetically active radiation (PAR) meter (Apogee MQ-200, Utah, USA) with three measurements per field replicate in each forest in all the collection days (described in next subsection, n = 32). Soil samples for nutrient analyses (n = 4 per forest) were collected down to 10 cm with a small shovel after removal of the litter. Samples were taken back to the University of Copenhagen (Denmark) and analysed for available phosphate, ammonium and nitrate (PO_4_^3−^, NH_4_^+^, NO_3_^−^). For this, 10 g of fresh soil was shaken on a rotation table shaker (at 200 rpm) with 50 ml of double distilled water (ddH_2_O) for 30 min and the filtered extracts were measured in a SEAL AA500 continuous flow analyzer (Seal Analytical, Mequon, USA). Total carbon (C) and total N were also measured by weighing approximately 12 mg of dried, ground soil into tin capsules and analyzed in an Euro EA elemental analyzer (EuroVector, Pavia, Italy) using orchard leaves (Leco, St. Joseph, USA) as standard. To assess the annual atmospheric deposition of ammonium (NH_4_^+^) and phosphate (PO_4_^3−^) in the forests, resin lysimeters (UNIBEST Ag Manager, USA) were placed between two 1 cm-thick glass wool disks inside 50 mL centrifuge tubes open in the top and bottom to allow water flow (Ackermann et al. 2012). Four replicates were placed into the moss carpet to capture deposition as well as throughfall and four replicates were hung with a string on trees at breast height to capture stemflow in each forest. Resins were extracted by adding 15 ml of 1 M HCl in 50 ml centrifuge tubes, shaking for 30 min on a table shaker, decanting the 15 ml extract into a clean tube, repeating the process 3 times (to obtain a final volume of 45 ml) and analyzed in the same flow analyzer mentioned above.

### Nutrient additions and sampling

We established a nutrient addition experiment in the two forest sites applying nutrient solutions in the following concentrations: (1) 100 kg N ha^–1^ yr^−1^ as NH_4_NO_3_; (2) 50 kg P ha^–1^ yr^−1^ as NaH_2_PO_4_.H_2_O (P_low); (3) high P addition as 100 kg P ha^–1^ yr^−1^ as NaH_2_PO_4_.H_2_O (P_high); (4) a combined treatment with N and P (NP) with 100 kg ha^–1^ yr^−1^ for both N and P; and (5) 0.007 kg Mo ha^–1^ yr^−1^ as Na_2_MoO_4_.2H_2_O. The addition rates were chosen to allow the comparison to other published studies in the field that used similar rates and also investigated the response in N fixation to nutrient additions in tropical ecosystems (Barron et al. 2009; Reed et al. 2011; Van Langenhove et al. 2021). In addition, we tested a higher rate of P, as moss species seem to respond differently to added P (Clasen et al. 2023), which could lead to a stronger response in nitrogenase activity. Nutrient solutions were diluted with tap water and sprayed on 50 × 50 cm^2^ plots containing mosses growing on the surface of fallen trunks near the ground as well as other parts of the plots (e.g. litter), with four replicates per treatment. The plots were located near to each other (ca. 50 cm distance), preferably with mosses growing in the same surfaces (i.e. fallen branches), to limit the differences in moss communities. Water only was sprayed onto the control plots, making up to a total of six treatments. The buffer space between the treatments was of 20–50 cm, a variation that is due to the often non-uniform distribution of mosses across the ecosystem. In addition, the nutrient additions were applied in the same order (CT—> Mo—> P_low—> P_high—> N—> NP) across all replicates and forests. The first nutrient additions were applied in November 2021. Part of the mosses were handpicked within the plots the following day (referred to as *time 1D*), aiming to evaluate a short-term response of one day after the nutrients were added. Samples were brought back to UCPH for N fixation measurements. A second fieldwork campaign was conducted in January 2023, in which another subset of the mosses were collected from all the same treatment plots before nutrients were applied once more (*time 1Y*), to assess a 1 year response in N fixation rates to the nutrients. This was followed by a second nutrient addition procedure and again another collection of an additional subset of mosses, on the next day (*time 1D*_*2*_*,* 1 day after the second nutrient addition). This allowed a direct comparison between both years, including a possible nutrient accumulation effect. For all sampling times, a mixed sample of the most abundant mosses found on surfaces near the soil of each forest were randomly handpicked from each of the plots, placed in plastic bags and kept cool and in the dark before and during transport (ca. 2 days). Upon arrival, mosses were immediately transferred to 20 ml transparent glass vials and placed in growth chambers (see conditions below).

### Nitrogen fixation

Nitrogen fixation was assessed via the acetylene reduction assay (ARA) (Hardy et al. 1973), which is an assessment of the nitrogenase enzyme activity. Prior to, and during incubations, samples were placed in a growth chamber at 20 °C, with a photoperiod of 12 h light/12 h dark, with a light intensity (photosynthetic active radiation, PAR) of 200 µmol photons m^−2^ s^−1^ for three days to allow acclimation. Mosses were regularly sprayed with ddH_2_O to ensure no moisture limitation. During the ARA incubations (ca. 6 h), the glass vials were sealed with a rubber septa and 2 ml of air was replaced with the same volume of acetylene gas (representing 10% acetylene in each vial) with sterile syringes. Samples were analysed with an Agilent 8890 Gas Chromatograph (GC) (Agilent, Santa Clara, USA) equipped with a J&W CarboBOND column (50 m × 0.53 mm × 5 µm) using a Flame Ionization Detector (FID) and coupled to an automatic headspace sampler. The GC was operated with helium as the carrier gas at a flow rate of 8.48 ml/min, a pressure of 10 psi and oven temperature at 60 °C. To account for any residual ethylene in the acetylene gas, empty vials with 10% acetylene gas only were analysed and the resulting ethylene concentration (ca. 0.05 mol/ml) detected was subtracted from the final ethylene area of all samples. Additionally, extra moss samples were incubated with plain air to check for any natural ethylene production from the mosses, which was not detected.

After N fixation assessments, samples were observed under an Olympus BX61 epifluorescence microscope (Olympus, Tokyo, Japan) to visually inspect the presence of cyanobacteria, which was confirmed in all samples with no clear difference observed between treatments. Measurements of pH and electric conductivity (EC) were assessed by adding 10 ml of ddH_2_O to each vial, shaking for 30 min and leaving them standing for the same period before measuring them with pH and EC meters (Mettler Toledo, Switzerland). With this method, we assessed the environmental pH and EC of the moss surface, which resembles the environment in which the N-fixing cyanobacteria living on the moss surface experiences. Samples were frozen and then freeze-dried and dry weight was recorded for the final calculations.

In addition, we calculated the biomass (dry weight) per unit area for the mosses in the laboratory and multiplied it by the ground cover percentage at each forest site. The ground cover percentages were estimated from pictures of the plots from the first year of sampling, with a resolution of 5 cm^2^, per 50 × 50 cm plots, based on the presence or absence of mosses. To attempt to upscale the potential contribution of N fixation associated with mosses for comparison between the different successional forests we used the theoretical factor of 3:1 (Hardy et al. 1973), similarly found in other montane cloud forests (Permin et al. 2022). This way we could convert our measurements of N fixation in the lab (ARA in nmol g dw^−1^ h^−1^) to N fixed (N kg ha^−1^ yr^−1^). As our main goal was to compare between different forest successional stages we assumed optimal activity throughout the year.

### Data analyses

To characterize the forests and test for differences between the two successional forest stages in nutrient content (soil and lysimeters) and environmental factors (e.g. temperature, light and moisture), T-tests were performed. To test for differences in N fixation rates between forest sites, we performed a two-way ANOVA with ethylene production associated with mosses from the control treatments as dependent variable, and ecotype and sampling time as independent factors. Treatment (nutrient additions) differences in ethylene production associated with the mosses collected from each nutrient addition plot, of each sampling time, for each forest type were tested with three-way ANOVAs. Normality of the data was checked via QQ-plots and Shapiro–Wilk tests, and when data was not normally distributed log-transformation was used to meet the criteria for the ANOVA tests. Tukey HSD tests evaluated the interaction of the variables with a 95% confidence level. Linear regression analyses between N fixation and moss pH or EC values, as well as soil nutrients and atmospheric deposition, were run to assess relationships between variables. To verify the effect size of each nutrient addition, we calculated response ratios as the natural log response ratio (lnRR) to the control treatment, where a positive lnRR (> 0) indicates that the nutrient addition promotes N fixation, and a negative lnRR (< 0) indicates a negative effect on N fixation rates (Dynarski and Houlton 2018). All analyses were performed on R 4.2.2 and RStudio v. 2023.12.1, and visualized with ggplot2 v. 3.4.3 (Wickham 2016) and myClim v. 1.0.1 packages (Man et al. 2023).

## Results

### Microclimate and nutrients in the forests

Mean soil and air temperatures were in general one degree higher in the natural regrowth forest (NR, ~ 16 °C) compared to the old growth forest (OG, ~ 15 °C) with the mean soil moisture also significantly differing between forest types (p < 0.0001, Table 1), with the NR slightly more humid (NR: 40 vs. OG: 38%). Higher light was measured in the NR (16.7 (± 2.6) vs. 8.8 (± 0.8) µmol m^−2^ s^−1^; p < 0.0001). Soil nutrients also differed between forest types, with higher nitrate and phosphate levels and C:N ratio found in the old growth forests. Atmospheric deposition of ammonium and phosphate did not statistically differ between forests or substrate. Therefore, combining the data from both lysimeters (hung and placed in the soil) the final mean values of 1.59 (± 0.4) and 1.46 (± 0.2) NH_4_ kg ha^−1^ yr^−1^, and 0.02 (± 0.001) and 0.03 (± 0.004) PO_4_ kg ha^−1^ yr^−1^ were recorded in the NR and OG forests, respectively (Table 1).

**Table 1:** Forest nutrients (in soil and in atmospheric deposition) and climate data recorded between December 2021 and January 2023 in two forest successional stages (natural regrowth, NR, and old growth, OG) in central Costa Rica

		Natural regrowth	Old growth
Soil nutrients µg g dw^−1^	PO_4_^+^-P	0.12 (± 0.01)^b^	0.26 (± 0.04)^a^
	NH_4_^+^-N	2.55 (± 0.5)	3.03 (± 1)
	NO_3_^−^-N	29.8 (± 4.3)^b^	34.2 (± 4.7)^a^
Total N/C %	N	1 (± 0.1)	1.2 (± 0.1)
	C	14 (± 2.2)^b^	19.5 (± 2.3)^a^
	C:N	14.1 (± 0.7)^b^	16.6 (± 0.6)^a^
Nutrients deposition kg ha^−1^ yr^−1^	T-PO_4_^+^	0.03 (± 0.003)	0.02 (± 0.003)
	T-NH_4_^+^	2.09 (± 0.8)	1.48 (± 0.13)
	S-PO_4_^+^	0.02 (± 0.001)	0.03 (± 0.009)
	S-NH_4_^+^	1.08 (± 0.4)	1.44 (± 0.41)
Temperature °C	Air 2 cm	16.3^a^	14.9^b^
	Air 15 cm	16.1^a^	14.9^b^
	Soil 6 cm	16.4^a^	15.1^b^
VSM %	Soil 15 cm	0.402^a^	0.383^b^
Light µmol photons m^−2^ s^−1^	PAR	16.7 (± 2.59)^a^	8.8 (± 0.83)^b^

OBS. Temperature and moisture (VSM: volumetric soil moisture) are shown as means of measurements taken every 15 min for 13 months with TMS-4 microclimate sensors (n = 266,214). Standard errors for those measurements are all lower than 0.0001. Light measurements were only taken during the sampling campaigns, near the mosses height, shown are means between those days (n = 32 per forest). Nutrients deposition assessed under the canopy (n = 4 per forest and substrate; T (throughfall) = lysimeters hung 1.5 m high and S (soil) = lysimeters placed in soil at moss height). Soil nutrients (n = 4 per forest)

Lowercase letters show significant statistical differences (*p* < 0.05) between forest types (T-test)

### Nitrogen fixation across forests

**Fig. 1:**
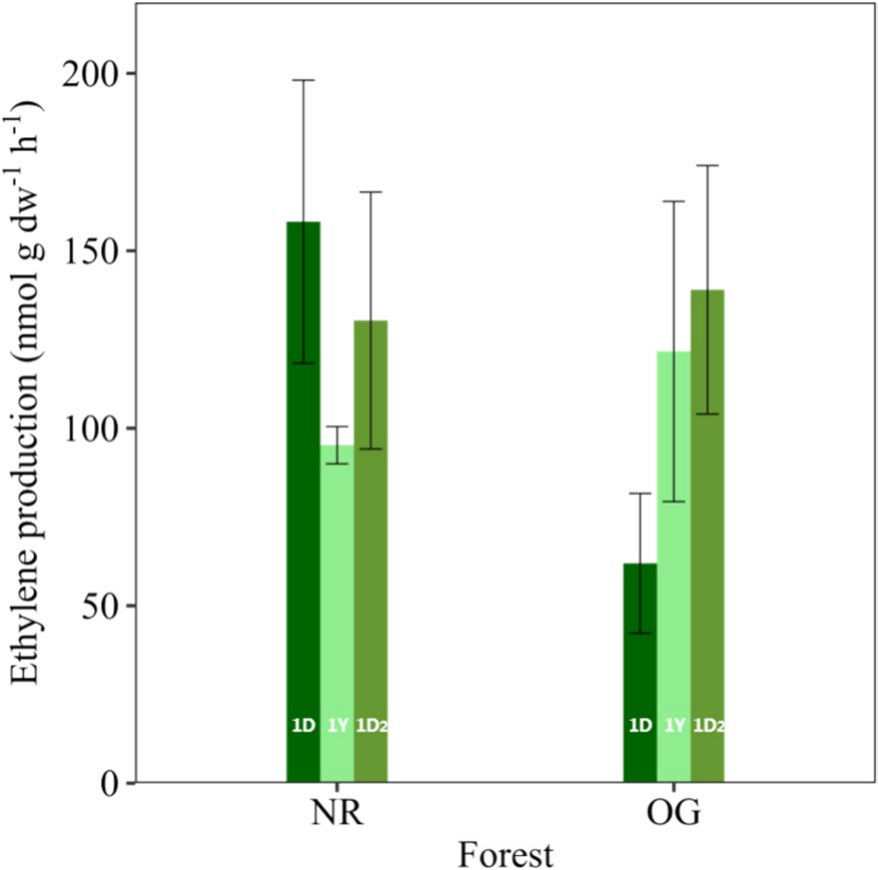
Mean ethylene production (nmol g dw^−1^ h^−1^, ± SE) associated with mosses from the control treatment across two different forest succession stages: natural regrowth (NR, n = 12) and old growth (OG, n = 12) forests. Different coloured bars represent the different sampling times for each forest type (dark green, 1D: 1 day after nutrient addition; light green, 1Y: 1 year after nutrient addition; and olive green, 1D_2_: 1 day after second nutrient addition). Sampling time is shown individually. No statistical differences were seen across time or forests, tested individually or combined

The mean ethylene production (N fixation) in association with mosses from the control plots across all time points were slightly higher in the NR forest (127.9 ± 18 nmol g^−1^ h^−1^) than the ones from the old OG forest (107.5 ± 20.2 nmol g^−1^ h^−1^). However, that difference was not statistically significant between forests, independent if sampling time was considered together or separately (Fig. 1). On the other hand, when considering the moss cover per plot in the two forests, the potential contribution of N fixation was slightly higher in the OG forests: 2.37 (± 0.58) kg N ha^−1^ yr^−1^, in comparison to the 2.03 (± 0.38) kg N ha^−1^ yr^−1^ estimated for the NR.

### Nutrient addition effects on moss-associated N fixation

**Fig. 2:**
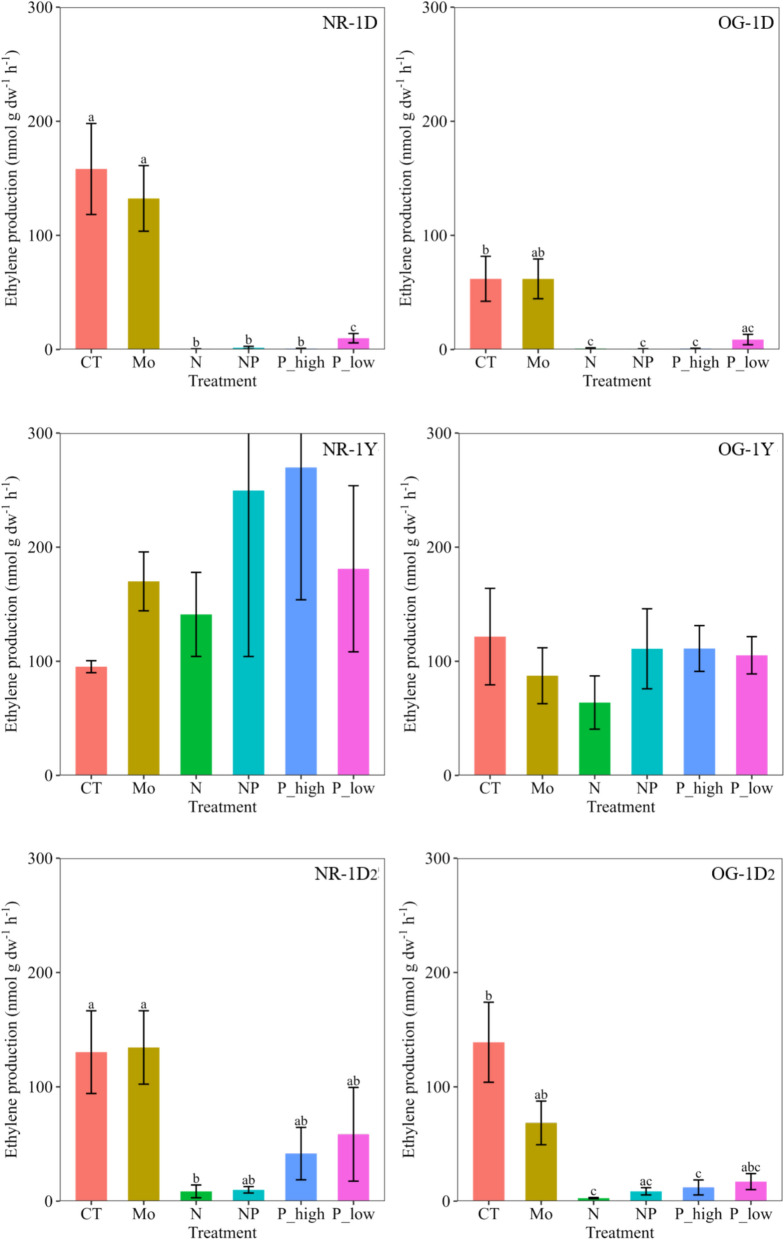
Mean ethylene production (nmol g dw^−1^ h^−1^, ± SE; n = 4 per treatment) associated with mosses in response to nutrient additions in two tropical montane cloud forests in Costa Rica in different successional stages (NR natural regrowth and OG old growth). Treatments are: control (CT), molybdenum (Mo), nitrogen (N), N and phosphorus (NP), and two levels of phosphorus (P_high and P_low) additions. Capital letters next to the forest type represent the different sampling time for each forest type (1D: 1 day after nutrient addition, 1Y: 1 year after nutrient addition; and 1D_2_: 1 day after second nutrient addition). Different lowercase letters on top of error bars (SE) indicate statistically significant differences between treatments (Tukey HSD test)

Treatment and sampling time had a highly significant effect on N fixation (p < 0.0001, F = 23.7 and 74.7, respectively), and differences between forest successional stages were also observed (p = 0.017, F = 5.7). Overall, except for Mo, the applied nutrients reduced N fixation rates, and those differences were observed for the two samplings performed one day after nutrient additions (p < 0.0001, F = 52.6 and 14 (*time 1D and 1D*_*2*_*,* respectively)), but not for the one year sampling (*time 1Y*) (Fig. 2).

The control treatments and the Mo additions maintained similar rates in both forests (Fig. 2). Treatments containing N rapidly reduced N fixation rates, but rates recovered within 1 year independent of forest type. In the P addition plots, a general negative effect was observed, with a slightly less prominent reduction occurring with the lower level-P addition. Besides, a less drastic negative effect of P additions was seen 1 day after the second nutrient addition (NR-1D_2_), with the mean N fixation rates almost three times higher (NR-1D_2_ P treatment mean rate: 50 (± 31.9) nmol g dw^−1^ h^−1^) than the mean rates seen in the OG forests (OG-1D_2_ P treatment mean rate: 14.5 (± 6.7) nmol g dw^−1^ h^−1^). The same tendency with large variation was observed in the samples collected one year after nutrient additions, where higher N fixation rates in the P plots of the NR forest were observed (NR-1Y P treatment mean rate: 225.4 (± 94.3) nmol g dw^−1^ h^−1^) compared to the OG forest (OG-1Y P treatment mean rate: 108.2 (± 18.2) nmol g dw^−1^ h^−1^). The NR forest also responded more strongly than the OG forest to NP additions after one year (NR-1Y: 249.6 (± 126) vs. OG-1Y: 111 (± 35) nmol g dw^−1^ h^−1^). Those differences in N fixation rates between forests for the 1 year sampling were confirmed by a two-way ANOVA (p = 0.003, F = 9.6), suggesting a higher general response of the NR sites to the nutrients.

**Fig. 3:**
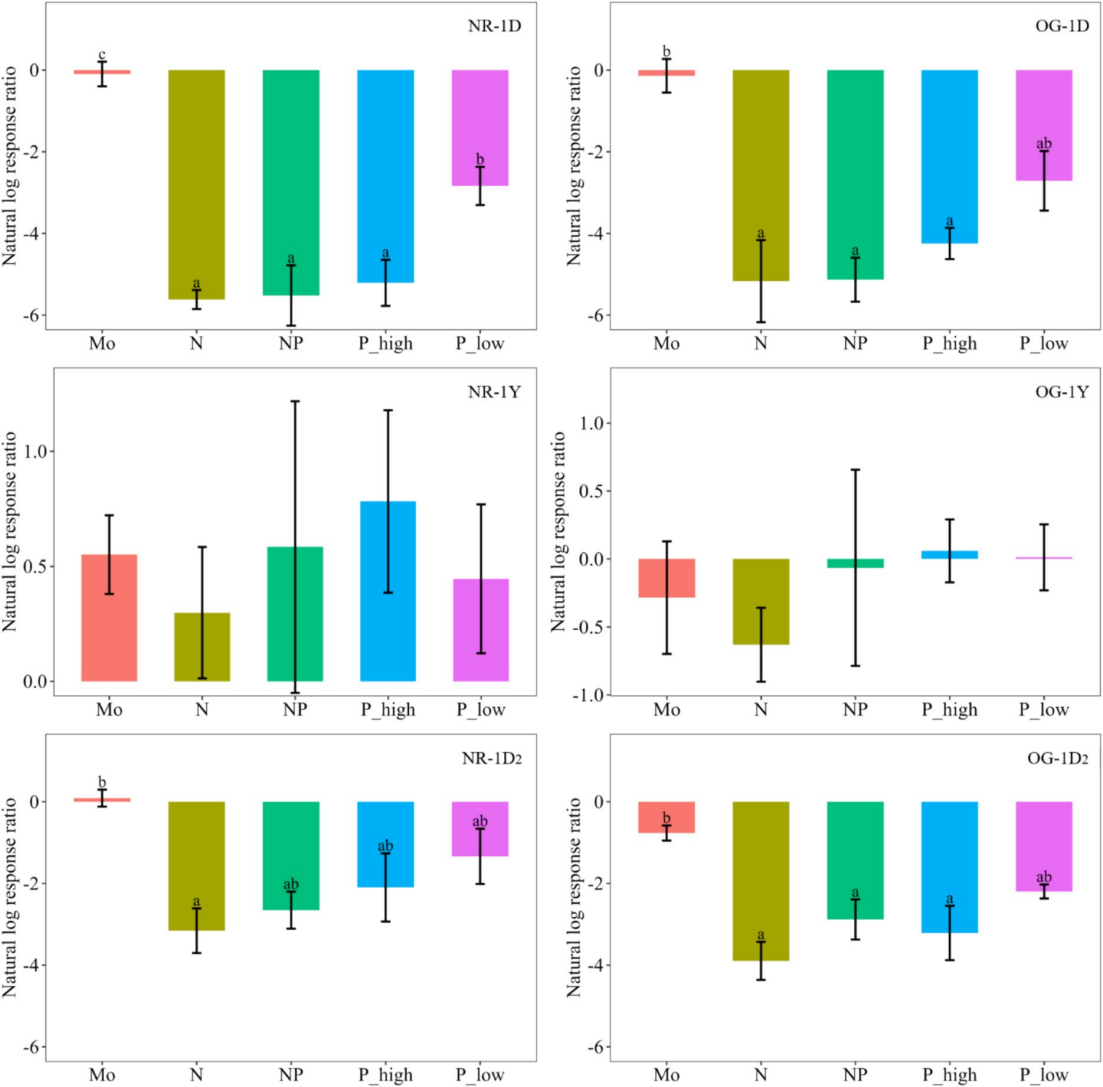
Response ratios (lnRR) relative to the control treatments of N fixation rates associated with mosses in response to nutrient additions. A positive lnRR (lnRR > 0) indicates promotion of N fixation rates and a negative lnRR (lnRR < 0) indicates a negative effect. Treatments are: control (CT), molybdenum (Mo), nitrogen (N), N and phosphorus (NP), and phosphorus in two levels (P_high and P_low). Two tropical montane cloud forests in Costa Rica under different succession stages are shown (NR: natural regrowth and OG: old growth). Capital letters represent the different sampling times for each forest type (A: 1 day after nutrients addition; B: 1 year after nutrients addition; and C: 1 day after second nutrients addition). Different small letters on top of error bars (SE) show statistically significant differences between treatments (Tukey HSD test). n = 4 per treatment

Focusing on the effect of the nutrient additions, we calculated response ratios relative to the rates from the control treatments (Fig. 3). The overall negative effect by the nutrient additions is seen in the response ratios for both sampling times right after the nutrients were added *(times 1D* and *1D*_*2*_). Specifically, all treatments plots from the time points right after the nutrients were added had reduced N fixation rates (lnRR < 0) in comparison with the controls, except the Mo addition treatment in the NR-1D_2_ (lnRR = 0.08). However, there was no statistical difference between the small positive response in the Mo treatment and the treatments containing P (Fig. 3 NR-1D_2_). On the other hand, we observed different responses for the one year sampling (*1Y*), with positive responses for all treatments in the NR forest (lnRR = 0.2 ~ 0.8). Opposite, we observed great variability in the response ratios for OG forest and no positive response could be confirmed (Fig. 3 OG-1Y).

### Moss pH and electric conductivity

Higher rates in N fixation were generally seen with moss pH ranging from 5.0 to 6.0 (Fig. S3). Moss pH varied across sampling times, even in the control samples, but there was no clear treatment effect. Nevertheless, some treatment differences were observed when sub-setting the data by time and forest type. For instance, in the OG forest, P additions increased moss pH (above 8.1) shortly after additions (p < 0.006; Table 2), but not in the NR forest. With the exception of Mo, nutrients generally increased EC, however significant differences between treatments were only confirmed for samples shortly after the first nutrient addition (1D) in both forests, but a larger significant increase was seen when N and P were added in combination (Table 2). Between forest types (OG vs. NR), pH was only significantly different in the Mo treatments at time 1D_2_ (p = 0.02), and EC values differed significantly in the control samples at time 1D_2_ (p = 0.04) (Table 2).

**Table 2:** Moss pH and electric conductivity (EC µS cm^−1^) across the six treatments (Control, molybdenum (Mo), nitrogen (N), N and phosphorus (NP), and phosphorus high and low (P_high and P_low)) in two forest succession stages (Old Growth and Natural Regrowth) across three sampling times (1D: one day after nutrient addition; 1Y: 1 year after nutrient addition; and 1D_2_: 1 day after second nutrient addition)

Treatment	Time	Old growth		Natural regrowth	
		pH	EC	pH	EC
Control	*1D*	7.16 (± 0.1)^b^	13.84 (± 3.8)^a^	7.51 (± 0.3)	34.03 (± 24.3)^a^
	*1Y*	5.70 (± 0.1)	90.78 (± 21.3)	5.88 (± 0.1)	82.48 (± 13)
	*1D*_*2*_	7.49 (± 0.1)^ab^	84.00 (± 22.5)^A^	7.77 (± 0.2)	25.61 (± 3.7)^B^
Mo	*1D*	7.10 (± 0.2)^ab^	18.58 (± 3.9)^a^	6.84 (± 0.2)	13.76 (± 3.8)^a^
	*1Y*	5.85 (± 0.1)	85.0 (± 18)	5.86 (± 0)	40.20 (± 9.7)
	*1D*_*2*_	6.91 (± 0.2)^a/B^	76.95 (± 26.1)	7.57 (± 0.1)^A^	27.38 (± 2.9)
N	*1D*	6.23 (± 0.3)^a^	218.18 (± 40.6)^a^	7.05 (± 0.7)	230.93 (± 45.7)^a^
	*1Y*	5.64 (± 0.1)	64.63 (± 25.8)	5.67 (± 0.1)	34.02 (± 14.2)
	*1D*_*2*_	7.14 (± 0.1)^a^	664.68 (± 173.8)	7.42 (± 0.2)	515.13 (± 205.6)
NP	*1D*	8.10 (± 0.3)^c^	664.63 (± 151.8)^b^	7.59 (± 0.1)	727.25 (± 133.3)^b^
	*1Y*	5.67 (± 0.2)	66.15 (± 18.6)	5.81 (± 0.2)	23.28 (± 5.8)
	*1D*_*2*_	7.59 (± 0.1)^ab^	735.0 (± 293.6)	7.60 (± 0.2)	179.5 (± 23.2)
P high	*1D*	8.46 (± 0.1)^c^	207.05 (± 15.3)^a^	8.2 (± 0.1)	236.93 (± 63.7)^a^
	*1Y*	5.84 (± 0.1)	67.73 (± 23.6)	5.81 (± 0.1)	38.1 (± 7)
	*1D*_*2*_	7.97 (± 0.1)^b^	429.25 (± 61.9)	7.80 (± 0.2)	356.33 (± 109.2)
P low	*1D*	8.28 (± 0.1)^c^	144.13 (± 23.3)^a^	8.07 (± 0.1)	169.05 (± 22.3)^a^
	*1Y*	5.88 (± 0.2)	67.08 (± 22.6)	5.79 (± 0.1)	35.72 (± 19)
	*1D*_*2*_	7.58 (± 0.3)^ab^	671.25 (± 240.1)	7.70 (± 0.2)	387.0 (± 121.3)

Lowercase letters show significant statistical differences (*p* < 0.05) between treatments and individual times (Tukey HSD test)

Capital letters show significant statistical differences (*p* < 0.05) between forest types

In the NR forest, a negative relationship between N fixation and moss pH for all treatments containing N and P (p < 0.04) was observed. In the OG forest, the same negative relationship was found for N fixation and the high P level treatment (p = 0.009) and the combined NP treatment (p = 0.007, Fig. S3), while no clear relationship was seen between the EC measurements and N fixation (Fig. S4). No clear links between N fixation and soil nutrients or atmospheric deposition were observed.

## Discussion

### Nitrogen fixation rates between forests

Nitrogen fixation rates associated with mosses in our study did not differ between the two investigated forests. We hypothesized (H1a) that higher moss-associated N fixation rates would be found associated with mosses from the early succession forest. This is based on the understanding that mature tropical forests generally accumulate N as they develop (Vitousek and Howarth 1991; Matzek and Vitousek 2003; Crews 2016), and these higher levels of N in the older forests could inhibit biological N fixation due to the high energetic cost of the process (Reed et al. 2011; Bellenger et al. 2020). Even though we measured higher soil inorganic N in the OG forest as predicted, this did not translate into lower N fixation rates. Additionally, when observing the samples under the microscope, we did not observe noticeable differences in cyanobacterial colonization between mosses of both forests, or between treatments (e.g. Fig. S1c, d), which is in line with the N fixation activity measurements.

Nonetheless, the lack of a negative effect of soil N on moss-associated N fixation is in line with previous research that suggested that N fixation associated with bryophytes could be decoupled from soil nutrients (Menge and Hedin 2009; Taylor et al. 2019) as a result of their non-vascular features. Furthermore, atmospheric N deposition can have a stronger impact on moss-associated N fixation compared to soil N pools (Gundale et al. 2011; Ackermann et al. 2012), allowing mosses to serve as indicators of anthropogenic N deposition (Salemaa et al. 2019). Atmospheric N deposition in both investigated forest sites were low and similar (< 1.6 kg NH_4_ ha^−1^ yr^−1^, Table 1), which likely contributes to the similar N fixation rates in the forest types. The potential contribution of N fixation associated with forest-floor mosses, considering the moss cover within the plots, was 2.37 (± 0.58) kg N ha^−1^ yr^−1^ in the OG forests, and 2.03 (± 0.38) kg N ha^−1^ yr^−1^ in the NR. These numbers are higher than the measured atmospheric N deposition in our plots, which should be considered with care as only NH4^+^ was measured in N deposition and actual N deposition that includes also nitrate and organic N may be higher (Cornell 2011; Bauters et al. 2018). However, the N fixation rates are in line with another study from a montane cloud forest in Peru that recorded rates of ~ 2 kg N ha^−1^ yr^−1^. Our estimates of N fixation are slightly higher in the OG than in the NR forest which is due to larger ground-cover of mosses within our investigated plots in the older forest (ca. NR: 39% vs. OG: 70%). Nonetheless, younger forests are often more N-limited (Menge et al. 2012; Powers and Marín-Spiotta 2017), leading to higher N fixation rates on mass basis, but the higher moss cover in the older forests, leading to higher N fixation on area basis, resulted here in similar net N fixation rates in the forest types. However, this contribution is likely to increase if epiphytic bryophytes are included (Alvarenga et al. 2024). A study investigating N fixation in tree epiphytic bryophytes vs. ground floor bryophytes also in Costa Rica, found higher rates associated within the epiphytic communities, which include some mosses (Markham and Fernández Otárola 2021). Besides, although we did not fully assess total moss biomass (only within our ground plots) we observed much higher coverage of epiphytic mosses in the OG forest than the NR. This is in line with another vegetation study in a nearby old growth forests within the same mountain range that reported higher biomass of bryophytes in the canopy compared to ground covers (Holz and Robbert Gradstein 2005). Nevertheless, the final contribution via moss-associated N fixation could potentially still be more significant in older mature forests after all, where more surface area is covered by epiphytic mosses.

### Nutrient addition effects on N fixation

Nitrogen fixation rates were immediately affected by the nutrient additions in both forests. For instance, the application of N to the mosses drastically reduced N fixation rates after one day in both sites as hypothesized (H2a). However, our second hypothesis also included a possible alleviation of negative effects in case P is applied in combination with N (H2b). We did not see that immediate alleviation, since the NP treatments also rapidly reduced N fixation rates. This suggests that the availability of N is a stronger factor controlling N fixation in association with mosses in these tropical forests.

We further hypothesized that P-only additions would promote N fixation in our study (H3). However, we observed mixed results, with P generally reducing N fixation rates as soon as it was added, and this immediate reduction was even more pronounced with the higher P levels. This immediate reduction could be associated with a possible osmotic effect influenced by the added nutrients and salt from the applied P solutions, as previously suggested by a study with mosses from a tundra ecosystem, which initially also had inhibited N fixation rates after high P additions (> 40 kg P ha^−1^ yr^−1^) (Rousk et al. 2017). Besides, although we did not directly measured moss biomass in the plots after the nutrient additions, we observed a visible decrease in the number of moss shoots in the treatments that received P in the second year, suggesting that they indeed experienced severe osmotic stress resulting from the elevated P additions. On the other hand, we observed a tendency with higher N fixation rates in the NR in the high P level plots after one year, as well as possible lower reduction after the second P addition when compared to the first. Although these results should be considered with care, these findings suggest (1) the addition of P could have increased photosynthetic activity of both cyanobacteria and mosses, and therefore, could have promoted the growth of the N-fixing community that fixes more N. This promotion via increased growth may only appear after months (Rousk et al. 2017). And (2) some of the remaining N-fixing community could have developed tolerance to the stress as well as possibly using more the added P more efficiently.

In addition, contrary to our 4th hypothesis, Mo additions did not promote N fixation in any forest type or time point. Positive effects of Mo on N fixation rates have been observed in studies with soil and litter in other tropical environments (Barron et al. 2009; Van Langenhove et al. 2021) as well as in subarctic mosses, both in the short and long-term (Rousk et al. 2017). We expected to see a fast response to the Mo additions as higher availability of Mo should rapidly promote nitrogenase synthesis translating to higher N fixation rates. However, this did not seem to be the case with tropical mosses as no evidence for Mo limitation was found in our study.

In our study, Mo treatments did not lead to any changes in electric conductivity, maintaining similar values as the ones found in the control plots of both forests (max. 90 µS cm^−1^). On the other hand, N and P additions immediately cause an increase in EC values, as high as seven times as the ones found in the controls of both forests. This indicates an imbalance in ions exchange that in the short term could have affected moss communities and therefore associated N fixation rates. Additionally, pH levels have been identified as indicators of moss-associated N fixation, with lower pH associated with decreased N fixation rates (Alvarenga and Rousk 2021). In our study, we also generally observed higher rates with pH levels between 5.0 and 6.0. However, N and P additions resulted in a general increase in pH levels, potentially contributing to the reduction in N fixation rates (Fig. S2). Nonetheless, further research on the effects of pH and EC on N fixation in association with mosses is still needed.

## Conclusion

Despite the potentially crucial role that moss-associated N fixation plays in ecological succession and ecosystem development, our knowledge about the controls of this process is still quite limited. This is especially true in very heterogeneous ecosystems like tropical forests, since the characterization and the dynamics of N in those habitats is still a big challenge. Moss-associated N fixation in tropical montane cloud forests seems to be little affected by the nutrient status (successional stage) of the forest. Molybdenum does not limit moss-associated N fixation in these forests, while rates can be rapidly impacted by events that could drastically increase N and P availability. However, N fixation rates can recover from elevated nutrient input within one year, with early successional forests potentially having a higher efficiency in utilising nutrients. Understanding the effects of nutrients on N fixation in different successional stages is necessary to foresee how different forests will respond to shifts in nutrients and climate. In the face of climate change and landscape transformations, this study further highlights the importance of investigating often overlooked forest components, such as mosses, which can act as valuable indicators of forest N status.

## Supplementary Information

Below is the link to the electronic supplementary material.

Supplementary file1 (DOCX 3759 KB)

## Data Availability

Data will be made available under reasonable request.
