# Supplementary material for: Rapid response of moss-associated nitrogen fixation to nutrient additions in tropical montane cloud forests with different successional stages
Source: Biogeochemistry. 2025 Jan 4;168(1):12. doi: 10.1007/s10533-024-01195-3 (PMC11700064; doi:10.1007/s10533-024-01195-3)
Supplement: Supplementary file 1 — Supplementary file1 (DOCX 3759 KB) [file 10533_2024_1195_MOESM1_ESM.docx]

SUPPLEMENTARY MATERIAL

**Rapid response of moss-associated nitrogen fixation to nutrient additions in tropical montane cloud forests with different successional stages**

Lina Avila Clasen*, Danillo Oliveira Alvarenga, Yinliu Wang, Rune Fromm Andersen, Kathrin Rousk


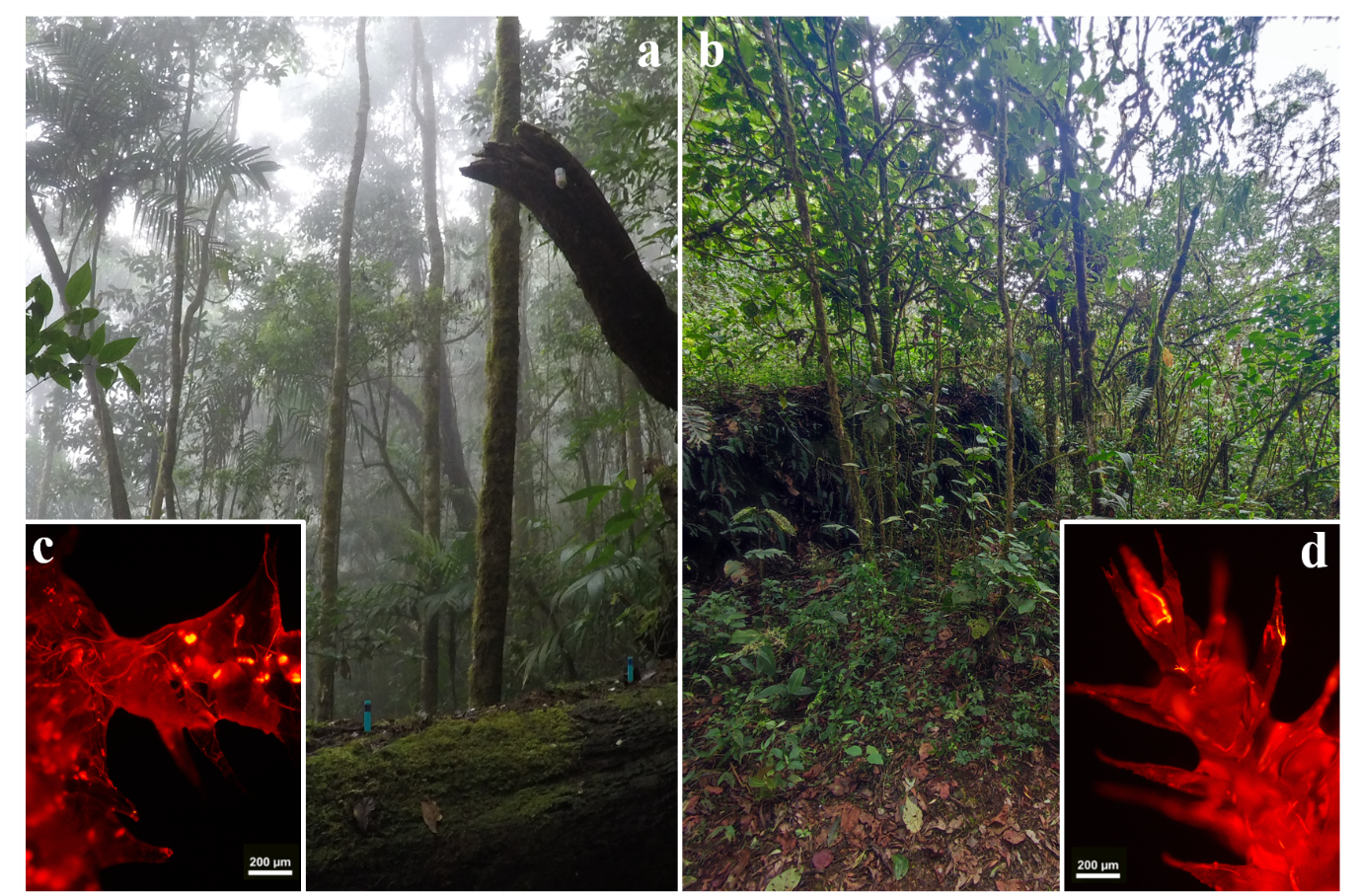


**Figure S1**. Tropical montane cloud forests in the Cloudbridge nature reserve in Costa Rica. a) Old growth forest; b) Natural regrowth forest. Red-glowing cyanobacterial filaments on top of mosses under green excitation filters of an Olympus BX61 fluorescence microscope are shown in c and d. Both mosses were collected from one of the phosphorus plots from each respective forest.


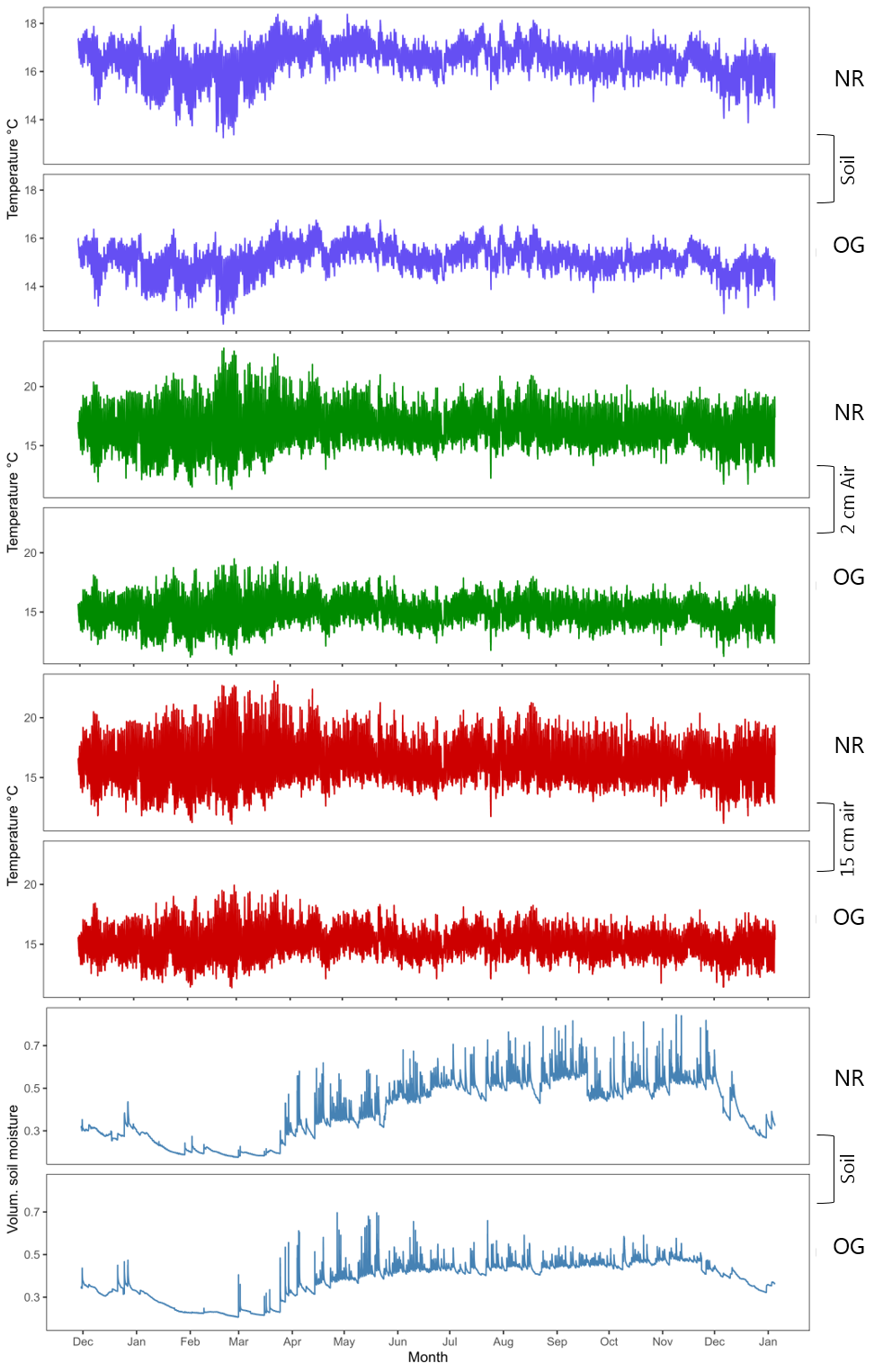


**Figure S2**. Climate data summary recorded between December-2021 and January-2023 for two tropical montane cloud forests in Costa Rica with TMS-4 microclimate sensors (TOMST, Prague, Czechia) in different successional stages (NR: natural regrowth and OG: old growth).


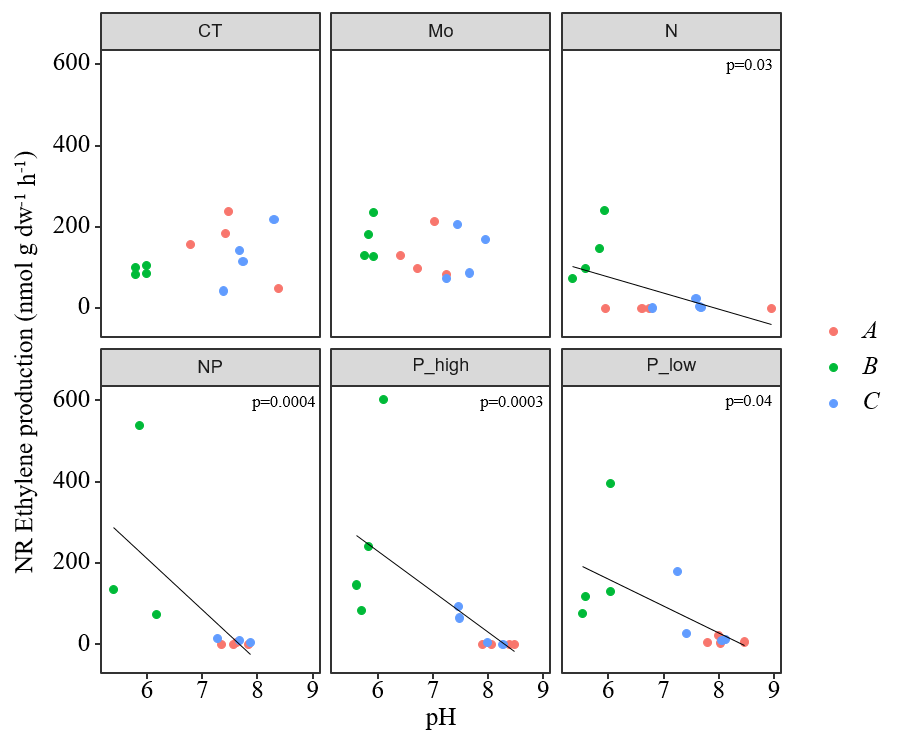


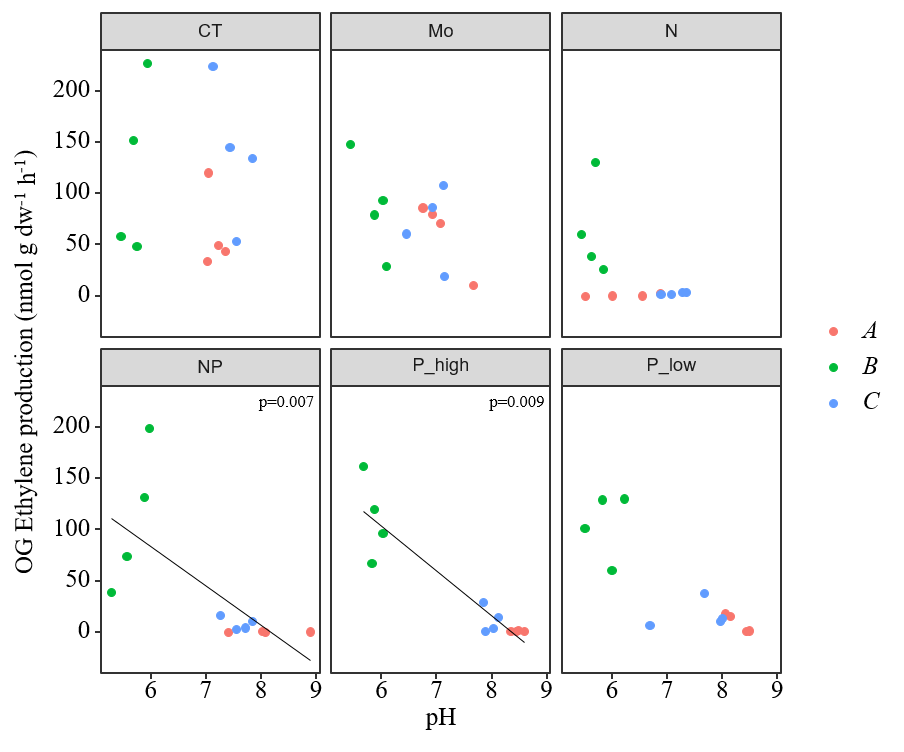


**Figure S3**. Linear regressions between ethylene production (nmol g dw^-1^ h^-1^) and pH for each of the six treatments (control (CT), molybdenum (Mo), nitrogen (N), N and phosphorus (NP), and high and low phosphorus concentrations (P_high and P_low)). Different colours show different sampling times (A: 1 day after nutrients addition (*1D*); B: 1 year after nutrients addition (*1Y*); and C: 1 day after second nutrients addition (*1D_2_*). Top plots: natural regrowth (NR) forest; bottom plots: old growth (OG) forest.

**
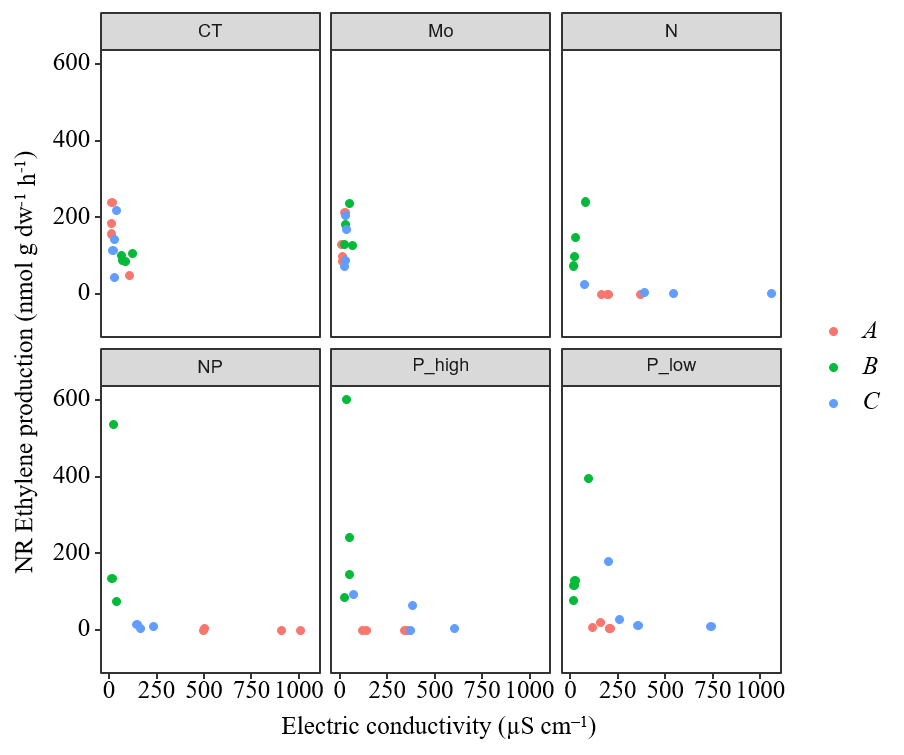
**

**
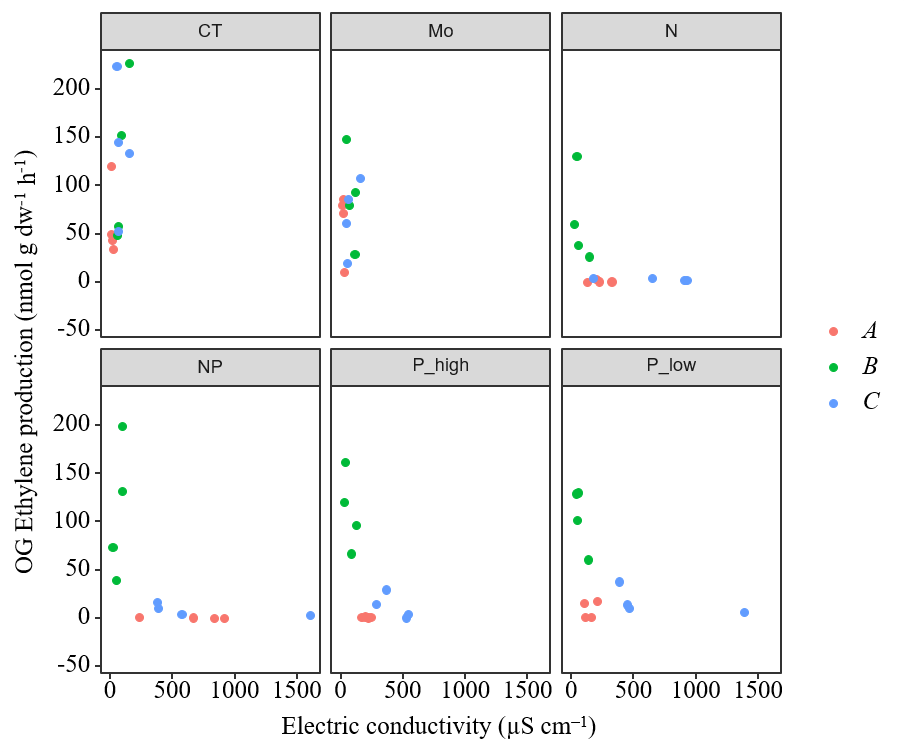
**

**Figure S4**. Linear regressions between ethylene production (nmol g dw-1 h-1) and electric conductivity (µS/cm) for each of the six treatments (control (CT), molybdenum (Mo), nitrogen (N), N and phosphorus (NP), and high and low phosphorus concentrations (P_high and P_low)). Different colours show different sampling times (A: 1 day after nutrients addition (*1D*); B: 1 year after nutrients addition (*1Y*); and C: 1 day after second nutrients addition (*1D_2_*). Top plots: natural regrowth (NR) forest; bottom plots: old growth (OG) forest.
